# Influence of playing position and laterality in centripetal force and changes of direction in elite soccer players

**DOI:** 10.1371/journal.pone.0232123

**Published:** 2020-04-23

**Authors:** Paulino Granero-Gil, Carlos D. Gómez-Carmona, Alejandro Bastida-Castillo, Daniel Rojas-Valverde, Ernesto de la Cruz, José Pino-Ortega

**Affiliations:** 1 Fitness Coach of PFC CSKA Moscow and Russian Football Union, Moscow, Russia; 2 Department of Physical Activity and Sport, Sport Science Faculty, Universidad de Murcia, San Javier, Spain; 3 Optimization of Training and Sport Performance Research Group (GOERD), Sport Science Faculty, University of Extremadura, Caceres, Spain; 4 Research Center of Sport and Health Diagnosis (CIDISAD), School of Human Movement Science and Quality of Life, Universidad Nacional, Heredia, Costa Rica; 5 Group in Updates for Sport Training and Physical Conditioning (GAEDAF), Sport Science Faculty, University of Extremadura, Caceres, Spain; Universidad Pablo de Olavide, SPAIN

## Abstract

The purpose of the present study was to: (a) assess centripetal force (CentF) and changes of direction (COD) in elite soccer players according to playing position (central defender, CD; lateral defender, LD; central midfielder, CM; lateral midfielder, LM; forward, FW), laterality (right-footed vs. left-footed) and field zone (central vs. lateral), and (b) analyze the relationship between anthropometric characteristics (age, weight, height, body mass and fat mass) and non-linear locomotion workload. Thirty professional soccer players (age: 26.57±5.56 years) were tracked during the 2017–2018 season during friendly, national and international matches (38 total games) using inertial measurement devices. CentF and COD were the variables extracted for analysis. A one-way ANOVA was used for playing position comparison, a t-test for laterality and field zone, and Pearson’s correlation coefficient to analyze relationships between anthropometric characteristics and dependent variables. There were differences by playing position in COD (556.33-to-412.18), R_20_COD (484.36-to-354.81) and R_60_COD (48.38-to-38.61) (*p* < .01; *ω*_*p*_^*2*^ = 0.03-to-0.05; CD>CM>LD>LM = FW); in COD_HIA_ (49.75-to-37.11), R_20_COD_HIA_ (16.04-to-9.11) and R_60_COD_HIA_ (10.64-to-9.11) (*p* < .01; *ω*_*p*_^*2*^ = 0.03-to-0.07; CM>FW>LM>CD = LD); in COD_SPRINT_ (14.56-to-8.40) and R_20_COD_SPRINT_ (3.29-to-1.40) (*p* < .01; *ω*_*p*_^*2*^ = 0.03-to-0.04; FW = LM = CM>CD = LD); and in CentF_MAX_ both in clockwise (992.04-to-902.09N) and counterclockwise (999.24-to-872.61N) directions (*p* < .02; *ω*_*p*_^*2*^ = 0.02-to-0.07; FW = CD>CM = LM = LD). The highest values of counterclockwise CentF were performed by left-footed players in the central zone (*p <* .001; *d* = 0.71-to-1.44) and clockwise CentF by right-footed players (*p <* .001; *d* = 0.04-to-0.55) in the lateral field zone. Moderate correlations were found between age, body mass and high intensity/sprints COD and repeated COD ability (*p <* .05; *r* = 0.235-to-0.383). Therefore, team staff should consider anthropometric characteristics, playing position, laterality and field zone to individualize training workload related to non-linear locomotion in soccer.

## Introduction

Soccer players cover a total distance of between 8 to 13 km [1]. Out of this total volume, the players need to transition between short high intensity efforts and longer periods of low activity, currently occurring more often due to the evolution of the game [2]. Approximately 12% of the total distance traveled is performed at sprint intensity with short efforts in time and distance [3]. In this analysis of soccer demands, many authors suggest that we should also take into account the acyclic activity of the player that includes changes in intensity, direction and movement patterns [4]. These are actions that are directly related to participation in the game, such as kicking or directing the ball, or duels with opponents to get possession, and that are not contemplated in the distance traveled, since there is no sensitive locomotion of the player, and consequently they cannot be measured with tracking systems (like global positioning systems, or local positioning systems) [5, 6].

Research into external workload during training and competition has focused on the variables mentioned above, in which linear actions predominate. This can be deduced by the small number of studies that address a curvilinear approach to player locomotion. Nevertheless, in some studies the triviality of linear actions and the importance of curvilinear locomotion have been argued [7–9]. In this respect, it has been shown that soccer players perform hundreds of changes of direction (COD) throughout the game [7]. Of note, approximately 85% of the actions executed at maximum velocity in elite teams consist of curvilinear sprints, that is to say the upright running portion of the sprint completed with the presence of some degree of curvature [10]. Furthermore, a curvilinear locomotor technique presents different kinetic and kinematic features [11, 12]. These differences can be reviewed in the work of Churchil et al. [13], but a key factor is trunk rotation, and consequently the neuromechanical requirements that this implies. For this reason, linear and nonlinear sprint performances embody different physical and technical capabilities and should be independently assessed and trained, for the same reason as for acyclic and non-sensitive locomotion, these actions cannot be measured with tracking systems.

Tracking systems are the most commonly used tool for professional soccer teams to monitor player loads patterns, mainly external load [14], due to the possibility of making estimations in a simple, portable and precise way [15]. Some tracking system models are integrated into inertial devices (IMU, Inertial Measurement Unit) which incorporate some sensors, such as accelerometers, that can measure non-locomotor actions, or gyroscopes, that can provide very relevant information about curvilinear locomotion: (i) the counting of events produced [16], (ii) indication of the degrees of rotation [17] and (iii) quantification of the angular speed of rotation [18]. Other variables that these devices can record are the estimation of the centripetal force (CentF) generated for the performance of the action (the combination of turn radius and locomotor speed) [19] and direction of locomotion. These variables related to curvilinear locomotion have not been addressed by the literature yet.

There are many studies that have analyzed the effect of independent variables like anthropometric measures, specific playing position, or the game field zone and laterality in soccer performance [2, 20–22]. Specifically, the most used dependent variables have been distance covered, speed, accelerations/decelerations and number of efforts above a predetermined threshold [5]. However, the dependent variables used in the present study have not been addressed yet by research and seem to be necessary for adopting a holistic approach to soccer performance analysis.

Therefore, the purposes of the present study were to: (a) assess CentF and COD in elite soccer players according to playing position (central defender, CD; lateral defender, LD; central midfielder, CM; lateral midfielder, LM; forward, FW), laterality (right-footed vs. left-footed) and field zone (central vs. lateral), and (b) analyze the relationship between anthropometric characteristics (age, weight, height, body mass and fat mass) and non-linear locomotion workload.

## Methods

### Design

In the current study, a longitudinal descriptive design with natural groups was followed [23] to describe the CentF and COD of elite soccer players during one season. En este sentido, Ninguna influencia respecto a la dinámica natural de la competición fue realiza, dando un tratamiento ecológico al estudio. In this regard, the study did not influence the natural dynamics of the competition in any way, thus giving it an ecological treatment. The data record refers to the 2017–2018 season and the matches played by an elite professional team in national (Russian Premier League and Russian Cup) and international (UEFA Champions League and UEFA Europa League) competitions. A total of 38 matches were recorded, of which 7 were played as locals, 17 as visitors and 14 as neutrals. In addition, 15 of the analyzed matches were in a national competition, 7 in an international competition and 16 were friendly matches. All the matches were played on outdoor natural grass and artificial turf pitches in accordance with FIFA rules. During the study, the analyzed team used a 4-4-2 formation.

### Participants

Thirty elite-level professional soccer players participated voluntarily in the present study (age: 26.57±5.56 years; height: 1.82±0.05 m.; body mass: 77.2±2.76 kg.; fat percentage: 8.44±1.08%; BMI: 22.31±1.32 kg/m^2^). For the characterization of the sample, the participating players were divided into five specific positions: (a) central defender (*n* = 4), (b) lateral defender (*n* = 5), (c) central midfielder (*n* = 8), (d) lateral midfielder (*n* = 5) and (e) forward (*n* = 8). To be included in the analysis the participants had to meet the following criteria: (i) the players had to play for the full duration of the game (~90min); (ii) not suffer an injury during the game; (iii) play in the same position throughout the game; (iv) be familiar with their playing position; and (v) goalkeepers were excluded from the analysis as they present a different internal and external workload profile from field players.

The study, which was conducted according to the Declaration of Helsinki, was approved by the Institution Review Board of the University of Murcia (Reg. Code: 2595/2019). Participants were informed of the risks and discomforts associated with testing and provided written informed consent.

### Procedures

Height was measured to the nearest 0.5 cm during a maximal inhalation using a wall-mounted stadiometer (SECA, Hamburg, Germany). Body mass and fat percentage were obtained with an 8-electrode segmental body composition monitor SC-240 model (TANITA, Tokyo, Japan). The accuracy for both variables has been analyzed in previous research and demonstrated acceptable results for estimating percentage of body fat mass when compared with DXA [24].

All team players wore inertial measurement devices (IMUs) (81x45x16 mm; 65gr.) with 10Hz GPS tracking system technology, four tri-axial accelerometers (±16, ±16, ±32 and ±400 G) and three 3D gyroscopes (±2000; ±2000 and ±4000 degrees/second) (WIMU PRO^™^, Almeria, Spain) in order to assess motion pattern data measurement during each match. The sampling frequency of inertial sensors can be configured between 10 and 1000 Hz. The gyroscope and accelerometer were configured at a sampling frequency of 100 Hz. The GNSS model used, that recorded at a sampling frequency of 18 Hz, has been validated previously [25] and has the FIFA certificate for use in professional soccer competitions. Besides, a previous evaluation was carried out with this model and has demonstrated very large to nearly perfect inter-unit reliability (ICC = 0.75–0.96) and good accuracy results (bias, counterclockwise = -2.19 N; clockwise = 1.75 N; typical error of measurement < 5%) for CentF variable during four different tracks (circle-6m radius, circle-9.15m radius, circle-12m radius, and combined track with zig-zag locomotion) and at different speeds (walking, high-intensity running and sprinting) (unpublished data). Only days when data collection occurred in conditions that were considered good for gathering valid and reliable GPS data [26] were included (2 matches were eliminated). During monitoring 12.8 ± 2.5 satellites were connected and Global Navigation Satellite System (GNSS) horizontal geometric dilution of precision (HGDOP) was 0.96±0.14, therefore almost ideal [27].

### Output variables

#### Non-linear locomotor performance

In the present study, the performance of non-linear locomotion was assessed through two principal variables: COD events and CentF generated in each event. The COD is considered as the specific event where one uses the “skills and abilities needed to change movement direction, velocity or modes” [28]. The CentF is considered as the force or the component of the force that acts on an object in movement on a curvilinear trajectory that is directed towards the center of curvature of the trajectory [19].

To calculate both variables, the information provided by the inertial sensors (accelerometer, gyroscope, magnetometer) is utilized and combined to obtain the relative position of the inertial device. This information along with the speed provided by the GPS and the player's weight are necessary to detect each COD event when the curvilinear locomotion lasts more than 800 milliseconds, and to calculate the CentF generated and the turning radius in each COD. Once this information is generated, the different CODs are classified based on their CentF, their angle of rotation or the speed at which they start.

Different COD variables were calculated from the detection of curvilinear locomotion with a duration of over 800 milliseconds: (a) CountCOD, number of total CODs performed in a match; (b) CountCOD_HIA_, number of total CODs performed in a match at high intensity (above 16 km/h); (c) CountCOD_SPRINT_, number of total CODs performed in a match at maximum intensity (above 21 km/h); (d) R_20_COD, number of total CODs performed with a recovery time of less than 20 seconds; (e) R_60_COD: number of total CODs performed with a recovery time of less than 60 seconds; (f) R_20_COD_HIA_: number of total CODs performed in a match at high intensity (above 16 km/h) with a recovery time of less than 20 seconds, (g) R_60_COD_HIA_, number of total CODs performed in a match at high intensity (above 16 km/h) with a recovery time of less than 60 seconds, (h) R_20_COD_SPRINT_, number of total CODs performed in a match at high intensity (above 21 km/h) with a recovery time of less than 20 seconds; and (i) R_60_COD_SPRINT_, number of total CODs performed in a match at high intensity (above 21 km/h) with a recovery time of less than 60 seconds.

Also, the following variables were extracted from the CentF detected in each COD: (a) -CentF_AVG_, average of the centripetal force generated by the player throughout the game when he turned counterclockwise; (b) +CentF_AVG_, average of the centripetal force generated by the player throughout the game when he turned clockwise; (c) -CentF_MAX_, maximum centripetal force generated by the player throughout the game when he turned counterclockwise; (d) +CentF_MAX_, maximum centripetal force generated by the player throughout the match when he turned clockwise; and (e) Difference (+% vs -%), average difference of centripetal force as a function of the direction of rotation.

#### Independent variables

Different contextual variables have been included in this research to identify their influence on CentF and COD performance as: (i) *playing position*, according to Dellal et al. (2011) divided into central defender (CD), lateral defender (LD), central midfielder (CM), lateral midfielder (LM) and forward (FW); (ii) *zone of the field*, dividing the pitch into central zone (CD, CM and FW) and lateral zone (LD and LM) [29]; and (iii) *laterality*, identified as the dominance of one leg and classified as left-footed and right-footed [30]. Also, different *anthropometric variables* have been recorded to analyze the effect on CentF and CODs dynamic such as age, body mass, height, fat percentage and body mass index.

### Statistical analysis

The descriptive statistics were calculated and reported as mean (M) ± standard deviations of the mean (SD) on each variable. Then, an exploratory analysis of the data was performed in order to confirm a normal distribution. A one-way ANOVA was used to detect differences among player positions. The Bonferroni post-hoc test was used to identify the source of any significant differences. A *t*-test was used to identify differences between laterality and the zone of the field. The magnitude of the differences was qualitatively interpreted using partial omega squared (*ω*_*p*_^*2*^) as follows: >0.01 small; >0.06 moderate and >0.14 large; and Cohen’s d (*d*) as follows: trivial (0–0.19), small (0.20–0.49), medium (0.50–0.79), or large (0.80 and above) [31].

Relationships between the CentF and COD performed with the anthropometrical variables were assessed using Pearson’s product-moment correlation (*r*). The magnitude of the correlation coefficients was deemed as very low (*r*^*2*^< 0.1), low (0.1 <*r*^*2*^< 0.3), moderate (0.3 <*r*^*2*^< 0.5), high (0.5 <*r*^*2*^< 0.7), very high (0.7 <*r*^*2*^< 0.9), nearly perfect (*r*^*2*^> 0.9) and perfect (*r*^*2*^ = 1) (Hopkins et al., 2009). Analyses were carried out using IBM SPSS Statistics (release 24.0; SPSS Inc., Chicago IL, USA) and figures were designed using GraphPad Prism (release 7; GraphPad Software, La Jolla CA, USA). Significance was established as p< 0.05 level.

## Results

Firstly, a differential analysis of COD performance variables in relation to playing position is shown in Table 1. Significant differences were shown in all CentF variables with low to moderate effect size (*ω*_*p*_^*2*^ = 0.02–0.07). Also, statistical differences were found in COD variables with low to moderate effect size (*ω*_*p*_^*2*^ = 0.02–0.07), except repeated COD ability 60-second rest at sprint (*p* = 0.07; *ω*_*p*_^*2*^ = 0.00). In relation to the individualized profile of COD: (a) central defenders presented higher demands in COD, R_20_COD and R_60_COD (CD>CM>LD>LM = FW); (b) central midfielders in COD_HIA_, R_20_COD_HIA_ and R_60_COD_HIA_ (CM>FW>LM>CD = LD); and (c) forwards in COD_SPRINT_ and R_20_COD_SPRINT_ (FW = LM = CM>CD = LD). Furthermore, with respect to the individualized profile of CentF, forwards and central defenders presented higher values of CentF_MAX_ both in clockwise and counterclockwise directions (*p* < .02; *ω*_*p*_^*2*^ = 0.02-to-0.07; FW = CD>CM = LM = LD).

**Table 1:** Comparison of centripetal force variables of match-play in relation to playing position.

	Central defender	Lateral defender	Central midfielder	Lateral midfielder	Forward	*p*	*F*	*ω*_*p*_^*2*^
	*M±SD*	*M±SD*	*M±SD*	*M±SD*	*M±SD*			
+ CentF_MAX_	988.63±141.66	942.86±213.17	902.09±160.35^e^	921.09±236.15	**992.04±337.49**	0.02	2.85	0.02
% + CentF	50.37±7.64	**51.35±5.80**^d^	50.41±6.07^d^	47.18±8.05	49.77±5.19	0.03	2.66	0.02
+ CentF_AVG_	205.60±10.10^e^	208.43±12.29^e^	208.95±15.18^e^	205.07±13.70^e^	**215.34±16.73**^abcd^	<0.01	5.99	0.05
- CentF_MAX_	-992.67±146.61^bcd^	-883.61±196.69^ae^	-873.42±187.10^ae^	-872.61±137.75^ae^	**-999.24±248.89**^**bcd**^	<0.01	8.82	0.07
%—CentF	49.63±7.64	48.65±5.80^d^	49.24±6.06^d^	**52.82±8.05**^bc^	50.23±5.19	0.02	2.90	0.02
- CentF_AVG_	-206.47±9.81	-207.34±17.90^e^	-206.17±14.92^e^	-210.41±34.82^e^	**-219.01±17.62**^abc^	< .001	8.84	0.07
Difference (+% vs -%)	0.74±15.28	2.69±11.60^d^	1.16±11.38^d^	**-5.63±16.11**^bc^	-0.46±10.38	0.02	2.88	0.02
COD	**556.33±150.59**^bde^	456.48±167.06^a^	490.66±203.77^e^	438.03±174.02^a^	412.18±178.59^ac^	<0.01	6.35	0.05
COD_HIA_ (>16 km/h)	37.11±14.99^c^	40.71±15.05^c^	**49.75±25.56**^ab^	39.75±20.98	44.54±21.15	<0.01	4.25	0.03
COD_SPRINT_ (>21 km/h)	8.40±4.28^e^	10.58±5.64^e^	12.19±9.87	11.14±9.51	**14.56±9.82**^ab^	<0.01	4.51	0.03
R_20_COD	**484.36±140.12**^de^	400.60±133.18	439.78±186.02^e^	372.46±148.29^a^	354.81±157.02^ac^	<0.01	6.59	0.05
R_60_COD	**48.38±15.42**^ce^	47.97±20.82^ce^	38.61±18.94^ab^	38.71±20.30	39.20±21.15^ab^	<0.01	4.37	0.03
R_20_COD_HIA_	9.11±5.36^ce^	11.23±6.21^c^	**16.04±10.23**^ab^	12.32±7.85	13.91±7.97^a^	<0.01	7.68	0.06
R_60_COD_HIA_	5.68±3.75^ce^	6.88±3.65^c^	**10.64±7.51**^ab^	7.86±6.10	8.89±5.47^a^	<0.01	8.21	0.07
R_20_COD_SPRINT_	1.40±1.51^e^	1.63±1.45^e^	2.57±3.07	2.25±2.86	**3.29±3.34**^ab^	<0.01	5.35	0.04
R_60_COD_SPRINT_	0.43±0.65	0.64±0.91	0.90±1.40	0.89±1.71	**1.00±1.23**	0.07	2.16	0.00

M: Mean; SD: Standard deviation; CentF: Centripetal force (+: clockwise, -: counterclockwise); COD: Changes of direction; RCOD: Repeated change of direction ability; p: p value; F: ANOVA F value; ω_p_^2^: partial omega squared; Statistical differences (p < .05) with ^a^central defender; ^b^lateral defender, ^c^central midfielder, ^d^lateral midfielder, ^e^forward; Bold represent the maximum values of each variable.

In Table 2, a comparative analysis was performed in relation to dominant leg and field zone. In the central zone, left-footed players presented higher values of centripetal force, both in clockwise and counterclockwise directions with respect to right-footed players (*p*<0.01; *d* = 0.71–1.44 *moderate* to *large*). In the lateral zone, no significant differences were found between left-footed and right-footed soccer players, except in +CentF_AVG_ with higher values in right-footed players (*p*<0.01; *d* = 0.55 *moderate*). Besides, only left-footed players presented differences in centripetal force performance, with higher values in the central zone with respect to the lateral zone.

**Table 2:** Comparison of centripetal force variables of match-play in relation to lower limb laterality and field zone.

Variables	Central zone		*p*	*t*	*d*	Lateral zone		*p*	*t*	*d*
	Right-footed	Left-footed				Right-footed	Left-footed			
	*M±SD*	*M±SD*				*M±SD*	*M±SD*			
+ CentF_MAX_	930.35±184.46	1263.47±614.19*	<0.01	-5.65	-0.71	**954.72±215.44**	906.82±200.06	0.22	1.24	0.23
+ CentF_AVG_	209.22±14.40	227.82±21.38*	<0.01	-4.86	-1.00	**211.02±13.51**	204.32±11.21	<0.01	2.97	0.55
- CentF_MAX_	-914.59±191.81	-1227.21±344.87*	<0.01	5.98	1.09	**-915.79±193.63**	-876.03±160.63	0.23	-1.19	-0.23
- CentF_AVG_	-208.57±14.61	-234.85±20.70*	<0.01	6.81	1.44	**-210.08±19.36**	-209.13±28.79	0.83	-0.22	-0.04
Difference (+% vs -%)	0.57±12.04*	0.40±9.71*	0.75	0.32	0.02	4.45±11.97	**-5.89±12.79**	<0.01	4.53	0.83

M: Mean; SD: Standard deviation; CentF: Centripetal force (+: clockwise, -: counterclockwise); p: p value; t: value of t-student of independent samples; d: Cohen’s d effect size;

*Statistical differences between central and lateral zone (p < .001); Bold represent the maximum values of each variable.

On the other hand, in the variable that analyzes the difference between the percentage of centripetal force generated in clockwise and counterclockwise directions, the central zone did not present differences between left-footed and right-footed players (*p*<0.75; *d* = 0.02), but differences were found in the lateral zone with a preference in right-footed players for the clockwise direction and in left-footed players for the counterclockwise direction (*p*<0.01; *d* = 0.83 *large*).

**Fig 1:**
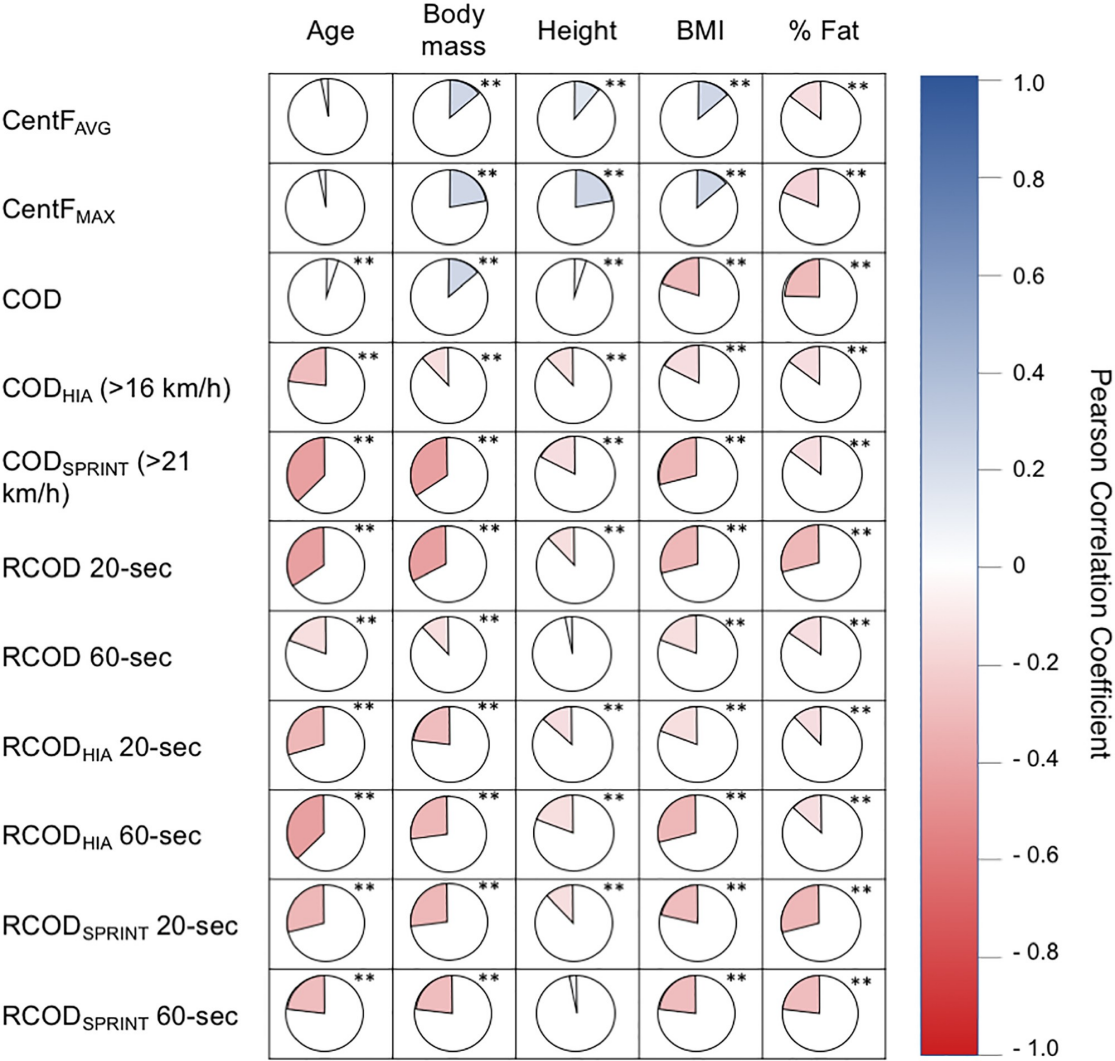
Pairwise Pearson correlation of the centripetal force and anthropometrical variables from official soccer match data. BMI: Body mass index (kg/m^2^); CentF: Centripetal force; COD: Change of direction; RCOD: Repeated change of direction ability. Significant correlation *(p < .05), **(p < .01).

The anthropometrical characterization of elite-level soccer players is shown in Table 3. Statistical differences in all anthropometrical variables were found with a moderate to large effect size (*ω*_*p*_^*2*^ = 0.11–0.45). CD were the soccer players who presented an older age, and higher weight and height, forwards presented the highest BMI, while CM were the players who presented the highest fat mass percentage (age: CD > LD > CM = LM = FW; weight: CD > LD = FW > CM = LM; height: CD > LD = FW > CM = LM; BMI: CD = LD = FW > CM = LM; fat mass: CM = LM = LD > CD = FW). In Fig 1, a correlational analysis was made between the anthropometric characteristics of the soccer players in relation to the CentF variables recorded by the inertial device. Low correlations were found between body mass, height and BMI with CentF_AVG_ (*r* = 0.131–0.162) and CentF_MAX_ (*r* = 0.163–0.233). Moderate inverse correlations were found between age and COD_HIA_ (*r* = 0.235), COD_SPRINT_ (*r* = 0.383), R_20_COD (*r* = 0.364), and R_60_COD_HIA_ (*r* = 0.353), R_20_COD_HIA_ (*r* = 0.284), R_20_COD_SPRINT_ (*r* = 0.268) and between body mass and COD_SPRINT_ (*r* = 0.312), R_20_COD *(r* = 0.301), R_60_COD_HIA_ (*r* = 0.269), R_20_COD_SPRINT_ (*r* = 0.288). Height, BMI and fat percentage obtained *very low* to *low* inverse correlations with the COD performance variables described previously (*r* = 0.124–0.278).

**Table 3:** Participants’ characterization and anthropometrical description.

	Central defender	Lateral defender	Central midfielder	Lateral midfielder	Forward	*p*	*F*	*ω*_*p*_^*2*^
Age (years)	**33.7±5.59**^bcde^	28.33±3.55^cde^	24.21±4.51	25.03±4.27	23.73±2.93	<0.01	66.58	0.39
Weight (kg)	**84.96±1.53**^bcde^	78.17±3.24^cd^	74.05±4.40	71.65±4.26	77.91±9.55^cd^	<0.01	43.56	0.29
Height (m)	**1.89±0.02**^bcde^	1.83±0.02^cd^	1.80±0.03	1.78±0.06	1.85±0.04^cd^	<0.01	83.40	0.45
BMI (kg/m^2^)	23.71±0.67^cd^	23.26±0.39^cd^	22.91±1.22	22.53±0.74	**23.93±1.99**^cd^	<0.01	13.50	0.11
Fat mass (%)	9.64±0.73	10.65±0.50^ae^	**11.01±1.19**^ae^	10.59±0.24^ae^	9.62±0.94	<0.01	32.02	0.23

BMI: Body mass index. Statistical differences (p < .05) with ^a^central defender; ^b^lateral defender, ^c^central midfielder, ^d^lateral midfielder, ^e^forward. Bold represent the maximum values of each variable.

## Discussion

During official matches, different acyclic activities were performed by the players that included changes of speed, direction of locomotion and movement patterns [1, 4]. These actions, that are directly related to participation in the game, are performed many times throughout the game [7]. Therefore, the purposes of the present study were to assess CentF and COD in elite soccer players according to playing position, laterality and field zone; and to analyze the relationship between anthropometric characteristics and non-linear locomotion workload.

### Playing position

COD is a recently used variable that could be affected by speed, neuromuscular and mechanical properties of the lower limbs, pivot leg reactive strength or technique [32]. In this study, differences were found by playing position. Central midfielders and forwards presented higher demands in repeated COD ability at sprint and high intensity that could be explained by their specific role and in-field zone of movement [7, 20, 33, 34], that usually requires non-linear trajectories due to the higher number of opponents who surround them. Additionally, the limited space in all directions as well as fewer moments spent standing still, require them to change direction not only more often but with greater intensity compared to lateral midfielders or lateral defenders [7].

On the other hand, the highest centripetal force generated in CODs was performed by central defenders who also recorded the greatest number of CODs. This indicates that the profile of non-linear locomotion by central defenders starts at a low speed (walking or jogging) and needs to achieve maximum acceleration with the purpose of covering the defensive line when forwards and wide midfielders of the opposing team overtake their teammates (central and lateral defenders) [7, 20, 33, 34]. Therefore, playing positions determine the specific CODs, centripetal force and repeated COD ability during competitive matches and should be considered when designing specific training workloads in non-linear displacements. The results discussed specify that central midfielders and forwards should be exposed during training to multiple tasks which require frequent and multiple changes of direction due to limited space and opposition from the opponent. Exposure to these preconceived scenarios during training may help to specifically prepare these players for the demands related to CODs in volume and intensity during competition. These tasks will not only prepare the player from a physical-technical but also tactical point of view where they will develop multiple responses to situations that vary in time and space. These short-term periods should be part of injury prevention protocols [35].

### Laterality

In the present study, laterality was evidenced by the fact that significant differences were reported in the players with right dominance vs. left dominance in the lateral zones, where they presented greater centripetal forces on the lateral side of their dominance. In this respect, previous research by Carey et al. [36] revealed a strong bias toward the use of the right foot in right-footed players and toward the use of the left foot in left-footed players across all soccer-related behaviors. Dominant foot use was highest for set-pieces (i.e., free kicks, penalty kicks, and corner kicks), dribbling, and passing (~85%). Hence, players utilize the non-dominant foot only when facing intense pressure from opponents.

This bias was reduced when the level of the players was higher, finding that two-footed players have an advantage for advancement to higher competitive levels [36]. For this reason, no differences were found in central zone players in the preference of COD direction, and a small difference was found only in lateral zone players. Therefore, for practical applications of training, this suggests that during the performance of curvilinear trajectories the inside leg works as a pivot and is usually the leg opposite to the direction of the turn or run [11] and should be trained specifically. The modification of the domination of CODs in the lateral zone could be produced by the tactics used by the team when interior locomotion predominates in the lateral zones.

Finally, as argued in the introduction section, CODs and the forces involved in high intensity actions are fundamental in soccer kinematics because these short-term actions may be a key parameter of soccer performance [35]. These actions usually open up clear scoring opportunities [37, 38] or allow the player to have a better position to shoot, unmark, pass or score [39]. The development of individualized training drills considering these external load variables should be addressed by the coaches and technical staff of professional teams in order to prepare not only their physical and technical abilities to face this type of actions but also to prevent potential injuries [40]. Besides, this type of variables may be included in future studies that analyze soccer performance or team players workload [28].

### Body composition

Age, body mass, height, fat mass and BMI of the players in this study were within the range previously reported in the literature for male elite soccer players (age: 22.1–28.1 years, body mass: 69.4–83.4 kg, height: 1.71–1.83, fat mass: 8.0–13.2, BMI: 20.8–22.9) [41]. Although previous studies showed the importance of anthropometric characteristics as an attribute for soccer performance in women elite players [42] and youth male players [43], few studies were found with the association between anthropometric characteristics and variables of soccer performance or players workload index in male elite players.

The present results show a directly significant relationship between body mass, height, and BMI with respect to CentF_MAX_ and CentF_AVG_ (*r* = 0.13–0.27; *p*<0.05). This could be explained because the weight of the subject is included in the calculation of the centripetal force generated [11]. Thus, it is possible that higher body mass, height, and BMI could contribute significantly to greater values of this kind of workload. However, an inversely significant relationship was reported between CentF_MAX_ (*r* = -0.19) and CentF_AVG_ (*r* = -0.15) with respect to the percentage of fat. In contrast, the percentage of fat showed lower significance with respect to body mass [41] in women players. Although there is a relationship between high BMI and higher CentF_MAX_ and CentF_AVG_, and an inverse relationship between percentage of fat with these variables, future studies should be carried out to confirm this finding due to the small lineal relation found between variables.

The previous information could help us to make practical recommendations to aid in the analysis and interpretation of body composition data in professional soccer. But in practical applications, possible variations in body composition could occur during the competitive season and must be taken into account, suggesting a need for continuous monitoring of this component of fitness across the entire season [44].

## Conclusions and practical applications

The anthropometrical characteristics of soccer players had a significant but small influence on the CODs and CentF performance, finding higher CentF demands in heavier and taller players and a lower number of CODs at low, moderate and high intensity in older, heavier and taller players. This aspect could suggest that: (i) older and taller players obtain lower values in this variable so they could not performed quick COD as easily as other lighter and younger players, although older players could and may replace this deficit with their experience, (ii) taller players need more CentF to change direction due to having a higher center of mass, and (iii) heavier players have a lower performance and greater risk of injury as they need more centripetal force to change direction due to greater body mass.

Players position and the zone of the field implied specific demands in CentF and CODs. In this respect, central midfielders and forwards presented the highest demands, and lateral zone players recorded asymmetry in centripetal force depending on the preferred dribbling direction.

Therefore, training plans and injury prevention sessions should be individualized to achieve the best performance in soccer players along the season. The COD actions should be introduced in training sessions and based on playing position, body composition and laterality, developing personalized training load. The volume and intensity of this type of actions, strategies and training tasks should be specific depending on these contextual factors, to facilitate the development of these fundamental skills for soccer performance. These contextual factors should be considered not only in technical and physical training, but also in tactics, injury prevention and decision-making related to when to return to play after an injury.

## Supporting information

S1 File (SAV)
